# Associations of Plasma Concentration Profiles of Dapagliflozin, a Selective Inhibitor of Sodium–Glucose Co-Transporter Type 2, with Its Effects in Japanese Patients with Type 2 Diabetes Mellitus

**DOI:** 10.3390/ph15020203

**Published:** 2022-02-07

**Authors:** Tetsuo Hayakawa, Ken-Ichiro Kato, Shinji Kobuchi, Kaede Kataoka, Toshiyuki Sakaeda

**Affiliations:** 1 Department of Diabetes Mellitus and Endocrinology, Tonami General Hospital, Toyama 939-1395, Japan; thayakawaendo@gmail.com (T.H.); katoken7@yahoo.co.jp (K.-I.K.); 2 Department Pharmacokinetics, Kyoto Pharmaceutical University, Kyoto 607-8414, Japan; kobuchi@mb.kyoto-phu.ac.jp (S.K.); ky16085@ms.kyoto-phu.ac.jp (K.K.)

**Keywords:** type 2 diabetes mellitus, dapagliflozin, plasma concentration, adherence, HbA1c, body weight

## Abstract

This study was conducted to evaluate the long-term plasma concentration profiles of dapagliflozin and its effects on the glycated hemoglobin (HbA1c) level, body weight, and estimated glomerular filtration rate (eGFR) in 72 Japanese outpatients with type 2 diabetes mellitus (T2DM) receiving metformin and a dipeptidyl peptidase-4 inhibitor. At baseline, HbA1c level, body weight, and eGFR were 6.9 ± 0.6%, 77.9 ± 13.5 kg, and 78.8 ± 20.7 mL/min/1.73 m^2^, respectively. A once-daily oral dose of 5 mg dapagliflozin was administered, and its trough plasma concentrations were evaluated at 1, 3, 6, 9, and 12 months. In this study, the patients with stable dapagliflozin concentrations were defined, based on a well-organized clinical trial, as those with average plasma concentrations of 2–5 ng/mL with a coefficient of variation <30%; these values were achieved if patients complied with their once-daily dosage. Multivariate analysis showed a significant decrease in the HbA1c levels among patients with stable concentrations (−0.6 ± 0.4%, *p* < 0.01), which was greater than the mean change among all 72 patients (−0.2 ± 0.5%, *p* < 0.01). The patients’ mean body weight also decreased (−2.3 ± 4.0 kg, *p* = 0.060). Average plasma concentrations ranged from 1.6 to 11.8 ng/mL; however, multivariate analysis indicated it was unrelated to the HbA1c-lowering effect. In conclusion, the long-term stability of plasma dapagliflozin concentration was important in lowering HbA1c level, and a once-daily oral dose of 5 mg was sufficient in achieving this effect.

## 1. Introduction

Sodium–glucose co-transporters (SGLTs), which are expressed in the proximal tubule, are involved in the renal reabsorption of glucose, and this process is mostly controlled by SGLT2 in the S1 segment [1,2]. SGLT2 inhibitors are a new class of agents used to treat type 2 diabetes mellitus (T2DM); they reduce glucose reabsorption and thereby reduce blood glucose without stimulating insulin release [1,2]. It is known that SGLT2 inhibitors exert metabolic, cardioprotective, and nephroprotective effects [3,4,5,6], although the underlying mechanisms remain unclear. Placebo-controlled studies have suggested that SGLT2 inhibitors reduce the risk of cardiovascular adverse events [7,8,9,10], and they are considered key drugs for treating cardiovascular diseases.

Dapagliflozin is a highly selective SGLT2 inhibitor used to treat T2DM [11,12]. A pooled analysis of safety data from phase IIb/III clinical trials showed that dapagliflozin has an acceptable safety profile [13]. The adverse effects of dapagliflozin include transient renal impairment, volume depletion, and genital and urinary tract infections [13]. The incidence of fracture and amputation was reportedly similar between the dapagliflozin and placebo/control groups [13]; these events are more specific for canagliflozin [10].

Dapagliflozin is rapidly absorbed, and peak plasma concentrations may be attained within two hours after single oral administration [12]. The systemic exposure, i.e., the area under plasma concentration–time curve (AUC) of dapagliflozin increased linearly from a dose of 0.1 to 500 mg, and it has a half-life of approximately 10 h [12]. There were no clinically meaningful differences in systemic exposure in terms of age, race, sex, body weight, food, or presence of T2DM [12]. Multiple dosing of 5, 25, and 100 mg for 14 days demonstrated that there is a dose-proportional exposure and dose-independent accumulation index in T2DM patients; however, the effects on fasting serum glucose and cumulative glucose excretion in the urine over 24 h were not proportional to the dose [11].

Little is known regarding the pharmacokinetic characteristics of dapagliflozin after long-term (>six months) administration, and much less information is available regarding the pharmacokinetic–pharmacodynamic relationship. Therefore, this study was conducted to evaluate the effects of long-term plasma concentration profiles of dapagliflozin on the glycated hemoglobin (HbA1c) level, body weight, and estimated glomerular filtration rate (eGFR) among Japanese outpatients with T2DM. The plasma dapagliflozin concentrations were determined at trough via liquid chromatography–tandem mass spectrometry (LC–MS/MS) at 1, 3, 6, 9, and 12 months after initiating treatment with dapagliflozin. Multivariate analysis was conducted, which included the average values of the concentrations, and the patient characteristics at baseline, such as sex, age, duration of T2DM, HbA1c level, body weight, eGFR, blood pressure, and comorbidities (diabetic nephropathy, hypertension, and hypercholesterolemia). In this study, the patients with stable dapagliflozin concentrations were defined. A randomized, double-blind, placebo-controlled, sequential, dose-ascending study using Japanese subjects (No. MB102010) described the plasma concentration–time profiles of dapagliflozin using a two-compartment model [14], and its trough concentration after long-term administration of 5 mg once-daily was estimated to be 2.4–3.4 ng/mL. Considering inter- and intraindividual variations, patients with stable concentrations were defined as those with average plasma concentrations of 2–5 ng/mL with a coefficient of variation (CV%) <30%; these values were achieved if patients complied with their once-daily dosage. 

## 2. Results

Seventy-two patients with T2DM were enrolled in the study. The patient characteristics at baseline are listed in Table 1. The male: female ratio was 52:20 and the mean age was 58.4 ± 10.6 years. The mean duration of T2DM was 10.8 ± 7.7 years. At baseline, the HbA1c level was 6.9 ± 0.6%, body weight was 77.9 ± 13.5 kg, eGFR was 78.8 ± 20.7 mL/min/1.73 m^2^, and systolic and diastolic blood pressures were 130 ± 16 and 73 ± 12 mmHg, respectively. Of the 72 patients, 27 had diabetic nephropathy, 53 had hypertension, and 54 had hypercholesterolemia.

Table 1 shows the results of univariate and multivariate analyses on changes in the HbA1c level after 12 months of treatment with once-daily oral dose of 5 mg dapagliflozin. The multivariate analysis showed that baseline HbA1c level, systolic blood pressure, and hypercholesterolemia influenced the HbA1c-lowering effect. However, none of variables investigated influenced the effects of dapagliflozin on body weight (Table S1) and eGFR (Table S2).

The average plasma dapagliflozin concentration was 1.6–11.8 ng/mL. This was not related to the HbA1c-lowering effect; however, the effect was greater among patients with stable concentrations (Table 1). Table 2 shows the changes in HbA1c level, body weight, and eGFR among patients with stable concentrations. The data for patients with poor adherence (those whose dapagliflozin concentrations went below the detection limit at one or more of the five sampling points) were also indicated as a reference. There was a significant change in the HbA1c level (−0.6% ± 0.4%, *p* < 0.01) in patients with stable concentrations, which was greater than the mean change for all 72 patients (−0.2% ± 0.5%, *p* < 0.01). There was also a mean decrease in body weight among these patients (−2.3 ± 4.0 kg, *p* = 0.060). There were no changes in the HbA1c level and body weight of patients with poor adherence. 

## 3. Discussion

Medication adherence is considered an important factor in determining clinical outcomes among patients with chronic diseases. For example, medication adherence has been suggested to be a contributor to cardiovascular diseases, as well as to the mortality and hospitalization of patients with T2DM [15,16,17]. However, the prevalence of poor adherence varies among reports, presumably due to the relatively low accuracy of the assessments and lack of a rational threshold for poor adherence [15,16]. Instead of using patient self-reports, reviewing pharmacy records, or counting pills, the ESC/ESH guidelines recently issued by the European Society of Cardiology and European Society of Hypertension recommend objective measurements of medication adherence, including biochemical analysis via LC–MS/MS [17]. In this study, the plasma dapagliflozin concentrations of outpatients were monitored over five visits. When the concentration was below the detection limit at one or more of these five points, the patient was considered to have poor adherence. As shown in Table 2, dapagliflozin had no effect on the HbA1c level and body weight among these patients, indicating the importance of medication adherence. Due to the small number of patients, the effects of poor adherence at different degrees could not be elucidated. Although biochemical analysis is not practical, this methodology is useful in identifying and providing direction for patients suspected to have poor adherence. A large, long-term study suggested that eGFR decreased in a dose-dependent manner after 1 week of treatment with dapagliflozin, then remained stable for 2 years, whereas eGFR slowly declined in the placebo group [18]. This implies that eGFR may be reduced in the patients with poor adherence; however, no such data was obtained in this study (Table 2).

In this study, patients with stable dapagliflozin concentrations were defined as those with average plasma concentrations of 2–5 ng/mL with a CV% < 30%; this label was given to patients that complied with the once-daily dapagliflozin dosing [14]. The HbA1c-lowering effect of dapagliflozin was greater among patients with stable concentrations relative to all 72 patients (Table 2). HbA1c is formed via non-enzymatic glycosylation reactions, and it reflects the ambient glucose level over the past 2–3 months [19,20]. Therefore, maintaining the glucose level seems to be important in lowering HbA1c levels. The average plasma dapagliflozin concentration varied from 1.6 to 11.8 ng/mL; however, this was not shown to be related to the HbA1c-lowering effect in T2DM patients, indicating that a once-daily oral dose of 5 mg dapagliflozin might be sufficient to reduce the HbA1c level.

There are substantial reports on the effects of long-term administration of dapagliflozin on clinical laboratory test parameters, including those concerning lipid profiles, and hepatic and renal function [21,22,23,24]. In this study, changes were observed in the serum uric acid, which decreased from 5.4 ± 1.2 to 5.0 ± 1.3 mg/dL (*p* < 0.01), and triglyceride, which decreased from 120 ± 48 to 107 ± 49 mg/dL (*p* = 0.028). The serum phosphate and magnesium levels increased from 3.3 ± 0.5 to 3.5 ± 0.5 mg/dL (*p* = 0.015) and from 1.8 ± 0.2 to 2.0 ± 0.2 mg/dL (*p* < 0.01), respectively. However, these changes were within the normal range, and their clinical significance remains unclear. Clinical studies on patients with abnormal values, and/or basic studies, may elucidate other potential effects of dapagliflozin.

Recently, the DAPA-HF Trial has demonstrated that dapagliflozin reduces the risk of worsening heart failure or death from cardiovascular causes, among patients with heart failure and reduced ejection fraction [25]. Furthermore, the DAPA-CKD Trial has proven that dapagliflozin reduces the risks of a composite of a sustained decline in the eGFR of at least 50%, end-stage kidney disease, and death from renal or cardiovascular causes, among patients with chronic kidney disease (CKD) [26]. It is noted that these conclusions have been drawn regardless of the presence or absence of diabetes. Cardiovascular risk factors include DM itself, hyperglycemia, arterial hypertension, and dyslipidemia [27]. The favorable results of the DAPA-HF Trial might be related to improvements in these factors. Patients with CKD have a high risk of adverse kidney and cardiovascular outcomes, and the main causes of death are cardiovascular in nature. Various underlying pathophysiological cascades, including vascular calcification, endothelial dysfunction, and oxidative stress, may be triggered by the accumulation of uremic toxins [28]. The nephroprotective effect of dapagliflozin observed in the DAPA-CKD Trial encourages further basic and/or clinical studies from a pathophysiological point of view. The dose of dapagliflozin used in these trials was 10 mg/day, twice that in this study. The average plasma concentrations varied from 1.6 to 11.8 ng/mL, indicating substantial interindividual differences in the pharmacokinetics. Clarification of factors influencing the pharmacokinetics might be helpful for identifying the patients exhibiting risk reductions in these trials.

This study has several limitations. First, the low number of patients reduced the statistical power of the variables (plasma concentration profiles and patient characteristics at baseline) identified as influencing the effects on HbA1c level, body weight, and eGFR after 12 months of treatment with dapagliflozin. Second, there were only 11 and 13 patients with poor adherence and stable concentrations, respectively. Future studies conducted on larger populations will help elucidate the effects of long-term plasma concentration profiles of dapagliflozin. Third, the clinical laboratory test parameters were within the normal range in most of the cases. Including patients with abnormal values may possibly help identify the pleiotropic effects of dapagliflozin. 

## 4. Materials and Methods

### 4.1. Clinical Study Design and Patients

Japanese outpatients that were diagnosed with T2DM at an early stage of diabetic nephropathy (urinary albumin-to-creatinine ratio of <30 mg/gCr), or with inadequate glycemic control, were enrolled in the study. Prior to the study, the patients were receiving daily metformin (1000 mg/day or more) and a dipeptidyl peptidase-4 inhibitor. A once-daily oral dose of 5 mg dapagliflozin after breakfast was prescribed for the patients. The medications were prepared as one dose per package. In Japan, the approved dose is 5 mg/day, although if insufficient for control, this can be increased to 10 mg/day; however, none of the patients in this study were prescribed that dose. Patient visits were scheduled to conduct clinical laboratory tests, vital sign monitoring, and other routine medical inquiries, at 1, 3, 6, 9, and 12 months after the prescription of dapagliflozin. At each visit, the patients were instructed to fast overnight. Blood samples were collected to determine the trough plasma concentration of dapagliflozin. Clinical laboratory tests included various metabolic and cardiovascular parameters. The eGFR was calculated using the serum creatinine level as eGFR (mL/min/1.73 m^2^) = 194 × [Serum creatinine]^−1.094^ × Age^−0.287^ × 0.739 (if female) [29]. The clinical study was conducted according to the guidelines of the Declaration of Helsinki and approved by the Ethics Committee of Tonami General Hospital (no. 26136) and Kyoto Pharmaceutical University (no. 16-07). Written informed consent was obtained from all participants prior to enrollment.

### 4.2. Determination of the Plasma Concentration of Dapagliflozin

The plasma concentration of dapagliflozin was determined via LC–MS/MS, following a previous study [30]. Briefly, 100 μL of acetonitrile containing 10 μL of internal standard working solution (5 μg/mL luseogliflozin) was added to 100 μL of plasma sample in a centrifuge tube. After vigorous mixing for 30 s, 1 mL of tert-butyl methyl ether was added to the tube. The mixture in the tubes was mixed vigorously for another 30 s, then centrifuged at 12,000 rpm for 15 min at 4 °C. The organic layer was then transferred into a 1.5 mL centrifuge tube and dried under nitrogen at 60 °C. The dry residue was reconstituted in 100 μL acetonitrile/10 mM ammonium acetate (50:50, *v*/*v*), and the reconstituted solution (30 μL) was injected into the LC–MS/MS system coupled with an API 3200 triple quadrupole mass spectrometer (Applied Biosystems/MDS Sciex, Foster City, CA, USA). The lower limit of detection for the analytes was 0.5 ng/mL in 100 μL of plasma sample. 

### 4.3. Statistical Analysis

All values were presented as the mean ± standard deviation (SD). Univariate analysis was conducted via Pearson’s correlation test, and the associations between the variables (plasma concentration profiles and patient characteristics at baseline) and the effects on the HbA1c level, body weight, and eGFR were evaluated. Multivariate analysis was then conducted using the variables, with *p* < 0.20, by univariate analysis, and the association was considered significant when *p* < 0.05. 

The data at baseline and after 12 months of treatment were compared using a Student’s paired *t*-test. The data of patients with poor adherence were compared with those with stable plasma dapagliflozin concentrations using a Mann–Whitney U-test. Differences between means were considered significant when *p* < 0.05. 

## 5. Conclusions

The long-term stability of plasma dapagliflozin concentration was an important factor in lowering HbA1c level, and a once-daily oral dose of 5 mg was sufficient to achieve this effect. 

## Figures and Tables

**Table 1:** Univariate and multivariate analyses on changes in HbA1c level after 12 months of treatment with 5 mg/day of dapagliflozin.

Variables		Univariate Analysis		Multivariate Analysis		
		r	*p*	B	β	*p*
Sex, male: female	52: 20	0.064	0.593			
Age, years	58.4 ± 10.6 ^a^	−0.101	0.397			
Body weight, kg	77.9 ± 13.5 ^a^	−0.015	0.901			
T2DM duration, years	10.8 ± 7.7 ^a^	0.047	0.697			
HbA1c level, %	6.9 ± 0.6 ^a^	−0.545	<0.001	−0.384	−0.456	<0.001
eGFR, mL/min/1.73 m^2^	78.8 ± 20.7 ^a^	0.034	0.780			
Systolic BP, mmHg	130 ± 16 ^a^	−0.295	0.012	−0.008	−0.258	0.008
Diastolic BP, mmHg	73 ± 12 ^a^	−0.071	0.553			
Diabetic nephropathy	27 of 72	0.052	0.669			
Hypertension	53 of 72	−0.058	0.626			
Hypercholesterolemia	54 of 72	−0.217	0.067	−0.232	−0.202	0.036
Average of Cp ^b^, ng/mL	4.7 ± 2.3 ^a^	0.071	0.630			
Stable Cp ^b,c^	13 of 72	−0.309	0.008	−0.287	−0.222	0.020

Variables with *p* < 0.20 in univariate analysis were used for multivariate analysis, and the results are presented as Pearson’s correlation: r, correlation coefficient; B, unstandardized estimated regression coefficient; β, standardized estimated regression coefficient. ^a^ The data are presented as the mean ± SD of 72 patients. ^b^ Cp: plasma concentration. ^c^ Based on a well-organized clinical trial [14], patients with stable concentrations were defined as those with average plasma concentrations of 2–5 ng/mL with a CV% < 30%.

**Table 2:** Effect of 12 months of treatment with dapagliflozin on the HbA1c level, body weight, and eGFR in patients with poor adherence and stable plasma dapagliflozin concentrations.

Group	N	Baseline	After Treatment	After Treatment—Baseline	*p*
HbA1c (%)					
Total	72	6.9 ± 0.6	6.7 ± 0.5	−0.2 ± 0.5	<0.01
Poor	11	6.8 ± 0.5	6.7 ± 0.7	−0.1 ± 0.4	0.298
Stable	13	7.1 ± 0.7	6.5 ± 0.5	−0.6 ± 0.4 *	<0.01
Body weight (kg)					
Total	72	77.9 ± 13.5	76.7 ± 13.9	−1.3 ± 2.6	<0.01
Poor	11	79.9 ± 7.1	78.7 ± 7.3	−1.2 ± 2.0	0.070
Stable	13	85.7 ± 12.4	83.4 ± 13.9	−2.3 ± 4.0	0.060
eGFR (mL/min/1.73 m^2^)					
Total	72	78.8 ± 20.7	75.2 ± 22.5	−3.6 ± 9.4	<0.01
Poor	11	95.5 ± 19.9	93.4 ± 19.0	−2.1 ± 9.9	0.524
Stable	13	74.1 ± 17.2	72.6 ± 18.3 *	−1.5 ± 5.1	0.329

The data at baseline and after 12 months of treatment were compared using a Student’s paired *t*-test, and the results are shown in the sixth column. Comparisons between the patients with poor adherence and stable concentrations were performed using a Mann–Whitney U-test, and statistically significant differences (*p* < 0.05) are marked with asterisks.

## Data Availability

Data is contained within the article and Supplementary Material.

## Supplementary Materials

The following supporting information can be downloaded at: https://www.mdpi.com/article/10.3390/ph15020203/s1, Table S1: Univariate and multivariate analyses on changes in body weight after 12 months of treatment with 5 mg/day of dapagliflozin; Table S2: Univariate and multivariate analyses on changes in eGFR after 12 months of treatment with 5 mg/day of dapagliflozin.
