# Supplementary material for: Associations of Plasma Concentration Profiles of Dapagliflozin, a Selective Inhibitor of Sodium–Glucose Co-Transporter Type 2, with Its Effects in Japanese Patients with Type 2 Diabetes Mellitus
Source: Pharmaceuticals (Basel). 2022 Feb 7;15(2):203. doi: 10.3390/ph15020203 (PMC8880045; doi:10.3390/ph15020203)
Supplement: Supplementary file 1 [file pharmaceuticals-15-00203-s001.zip › pharmaceuticals-1586138-supplementary.pdf]

Table S1 Univariate and multivariate analyses on changes in body weight after 12 months of treatment with 5 mg/day of dapagliflozin

Variables		Univariate analysis		Multivariate analysis		
		r	*p*	B	β	*p*
Sex, male: female	52: 20	0.104	0.386			
Age, years	58.4 ± 10.6 a)	−0.087	0.469			
Body weight, kg	77.9 ± 13.5 a)	0.059	0.625			
T2DM duration, years	10.8 ± 7.7 a)	0.169	0.157			
HbA1c level, %	6.9 ± 0.6 a)	0.084	0.480			
eGFR, mL/min/1.73 m2	78.8 ± 20.7 a)	0.017	0.892			
Systolic BP, mmHg	130 ± 16 a)	−0.135	0.259			
Diastolic BP, mmHg	73 ± 12 a)	−0.189	0.112	−0.035	−0.157	0.181
Diabetic nephropathy	27 of 72	0.134	0.264			
Hypertension	53 of 72	0.104	0.387			
Hypercholesterolemia	54 of 72	0.202	0.089	1.045	0.178	0.132
Average of Cp b), ng/mL	4.7 ± 2.3 a)	−0.156	0.288			
Stable Cp b, c)	13 of 72	−0.197	0.098	−1.327	−0.201	0.085

Variables with $p < 0.20$ by univariate analysis were used for multivariate analysis, and the results were presented as Pearson's correlation; r: correlation coefficient, B: unstandardized estimated regression coefficient, β: standardized estimated regression coefficient.

a) The data presented were the mean ± SD of 72 patients.

b) Cp: plasma concentration

c) Based on a well-organized clinical trial [14], patients with stable concentration were defined as those with average plasma concentrations of 2–5 ng/mL with a CV% < 30%.

Table S2 Univariate and multivariate analyses on changes in eGFR after 12 months of treatment with 5 mg/day of dapagliflozin

Variables		Univariate analysis		Multivariate analysis		
		r	*p*	B	β	*p*
Sex, male: female	52: 20	0.121	0.318			
Age, years	58.4 ± 10.6 a)	−0.014	0.910			
Body weight, kg	77.9 ± 13.5 a)	0.122	0.313			
T2DM duration, years	10.8 ± 7.7 a)	−0.228	0.058	−0.259	−0.212	0.072
HbA1c level, %	6.9 ± 0.6 a)	0.014	0.909			
eGFR, mL/min/1.73 m2	78.8 ± 20.7 a)	−0.028	0.819			
Systolic BP, mmHg	130 ± 16 a)	−0.169	0.163	−0.092	−0.152	0.196
Diastolic BP, mmHg	73 ± 12 a)	−0.009	0.940			
Diabetic nephropathy	27 of 72	−0.085	0.488			
Hypertension	53 of 72	−0.231	0.054	−4.272	−0.200	0.091
Hypercholesterolemia	54 of 72	−0.006	0.959			
Average of Cp b), ng/mL	4.7 ± 2.3 a)	0.032	0.833			
Stable Cp b, c)	13 of 72	0.102	0.403			

Variables with $p < 0.20$ by univariate analysis were used for multivariate analysis, and the results were presented as Pearson's correlation; r: correlation coefficient, B: unstandardized estimated regression coefficient, β: standardized estimated regression coefficient.

a) The data were presented as the mean ± SD of 72 patients.

b) Cp: plasma concentration

c) Based on a well-organized clinical trial [14], patients with stable concentration were defined as those with average plasma concentrations of 2–5 ng/mL with a CV% < 30%.
